# Mitochondrial protein sulfenation during aging in the rat brain

**DOI:** 10.1007/s41048-018-0053-3

**Published:** 2018-04-19

**Authors:** Xiaorong Yang, Jinzi Wu, Siqun Jing, Michael J. Forster, Liang-Jun Yan

**Affiliations:** 1 Department of Pharmaceutical Sciences, UNT System College of Pharmacy, University of North Texas Health Science Center, Fort Worth, TX 76107 USA; 2 Department of Physiology, National Key Disciplines, Key Laboratory for Cellular Physiology of Ministry of Education, Shanxi Medical University, Taiyuan, 030001 China; 3 College of Life Sciences and Technology, Xinjiang University, Urumqi, 830046 China; 4 Center for Neuroscience Discovery, Institute for Healthy Aging, University of North Texas Health Science Center, Fort Worth, TX 76107 USA

**Keywords:** Two-dimensional polyacrylamide gel electrophoresis (2D-PAGE), Brain aging, Mitochondria, Sulfenation, Oxidative stress

## Abstract

There is accumulating evidence that cysteine sulfenation (cys-SOH) in proteins plays an important role in cellular response to oxidative stress. The purpose of the present study was to identify mitochondrial proteins that undergo changes in cys-SOH during aging. Studies were conducted in rats when they were 5 or 30 months of age. Following blocking of free protein thiols with *N*-ethylmaleimide, protein sulfenic acids were reduced by arsenite to free thiol groups that were subsequently labeled with biotin-maleimide. Samples were then comparatively analyzed by two-dimensional Western blots, and proteins showing changes in sulfenation were selectively identified by mass spectrometry peptide sequencing. As a result, five proteins were identified. Proteins showing an age-related decrease in sulfenation include pyruvate carboxylase and pyruvate dehydrogenase; while those showing an age-related increase in sulfenation include aconitase, mitofilin, and tubulin (α-1). Results of the present study provide a general picture of mitochondrial protein sulfenation in brain oxidative stress and implicate the involvement of protein sulfenation in overall decline of mitochondrial function during brain aging.

## **INTRODUCTION**

Brain aging is associated with a shift from cellular redox regulation to oxidative damage (Rebrin *et al*. 2007; Sohal and Forster 2007). This redox shift to a more oxidative status during aging is thought to be responsible for oxidative modification of reactive protein cysteine (cys) residues that are involved in redox signaling upon oxidative stress (Cai and Yan 2013; Ying *et al*. 2007). A major product of protein cys oxidation is a protein sulfenic acid (PSOH), a reversible oxidative modification of a protein thiol group (Heppner *et al*. 2017; Poole *et al*. 2004). Sulfenic acid is a central intermediate during cys oxidation and can be induced by hydrogen peroxide, alkyl hydroperoxides, and peroxynitrite (Poole *et al*. 2004). Although long considered highly reactive and unstable, accumulating evidence has indicated that stabilized sulfenic acids do exist and play a key redox regulatory role in a growing number of proteins (Boschi-Muller *et al*. 2008; Claiborne *et al*. 2001; Denu and Tanner 2002; Kaiserova *et al*. 2006; Shetty *et al*. 2007; Turell *et al*. 2008; Wood *et al*. 2003; Yeh *et al*. 1996). Moreover, the biological and pathological significance of PSOHs has been further demonstrated by reports that PSOH formation is required for T-cell activation (Michalek *et al*. 2007) and that many proteins in cancer cells contain sulfenic acids (Leonard *et al*. 2009; Seo and Carroll 2009).

**Fig. 1:**
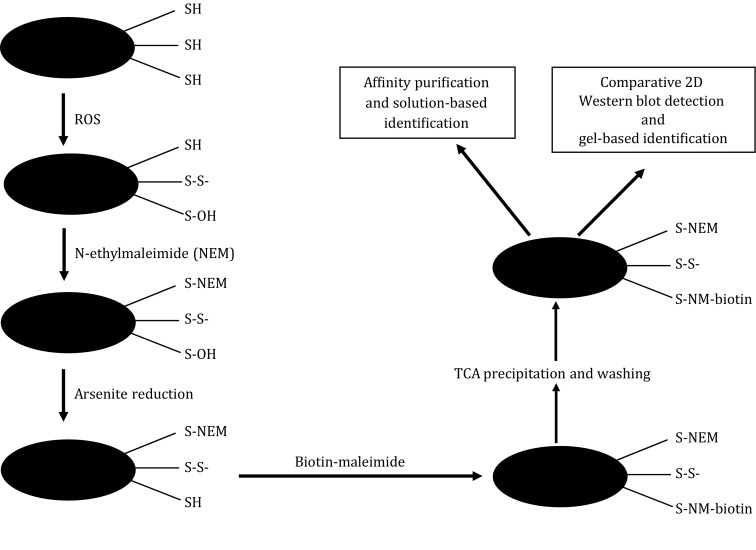
Scheme outlining the procedure of arsenite reduction/biotin switch used for detection and identification of proteins that underwent sulfenation in rat brain mitochondria. All available free protein thiol groups were blocked with *N*-ethylmaleimide (NEM) in the presence of 1% SDS that facilitates the access of free thiols by the alkylating reagent. Excessive NEM was removed by gel filtration using PD-10 columns. The resulting protein solution was treated with sodium arsenite in the presence of biotin-maleimide. Following precipitation with 10% TCA and washing with ethyl acetate/ethanol, protein pellet was dissolved in corresponding buffers for 2D Western blot detection or for affinity purification

The purpose of the present study was to identify mitochondrial proteins that undergo changes in cys sulfenation (cys-SOH) during brain aging and oxidative stress. Mitochondria were used in this study because mitochondrial oxidative stress has been associated with brain aging (Sohal and Forster 2007) and a number of neurodegenerative disorders including Parkinson’s and Alzheimer’s disease (Keating 2008). In this study, rats aged at 5 or 30 months were used. Following mitochondria isolation and blocking of free protein thiols with *N*-ethylmaleimide (NEM), sulfenic acids in proteins were captured by an arsenite-specific reduction/biotin switch method (Saurin *et al*. 2004), whereby a sulfenic acid was reduced back to a free thiol group that was subsequently labeled with biotin-maleimide (Fig. 1). Samples were then analyzed by comparative two-dimensional (2D) Western blots and sulfenated proteins were identified by mass spectrometry (MS) peptide sequencing. Results of the present study suggest that mitochondrial PSOHs play a role in brain aging.

## **RESULTS**

### **Specificity of the arsenite reduction/biotin switch method in conjunction with reducing gel electrophoresis**

**Fig. 2:**
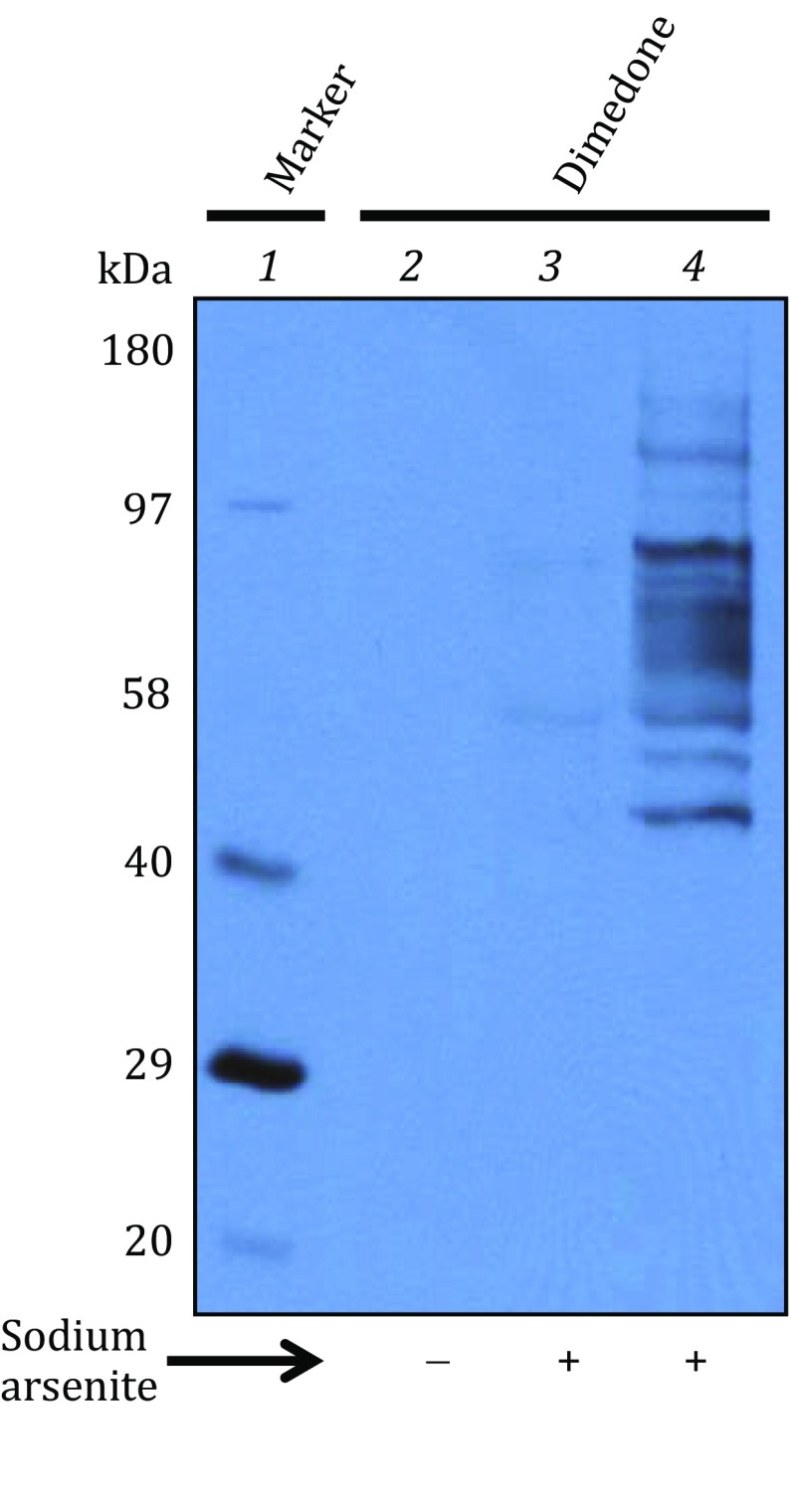
A representative 1D Western blot demonstrating a complete removal of biotin-maleimide by the procedure of TCA precipitation and organic solvent washing. *Lane 1*: biotin-conjugated protein markers (Bio-Rad); *Lane 2*: biotin labeling of the protein mixture in the absence of arsenite; *Lane 3*: biotin labeling of the protein mixture in the presence of arsenite following dimedone treatment; *Lane 4*: biotin labeling of the protein mixture in the presence of sodium arsenite. Samples (20 μg/lane) loaded onto the gel were derived from mitochondria that underwent *in vitro* oxidative stress in the presence of succinate and antimycin A as previously described (Yan *et al*. 2008)

**Fig. 3:**
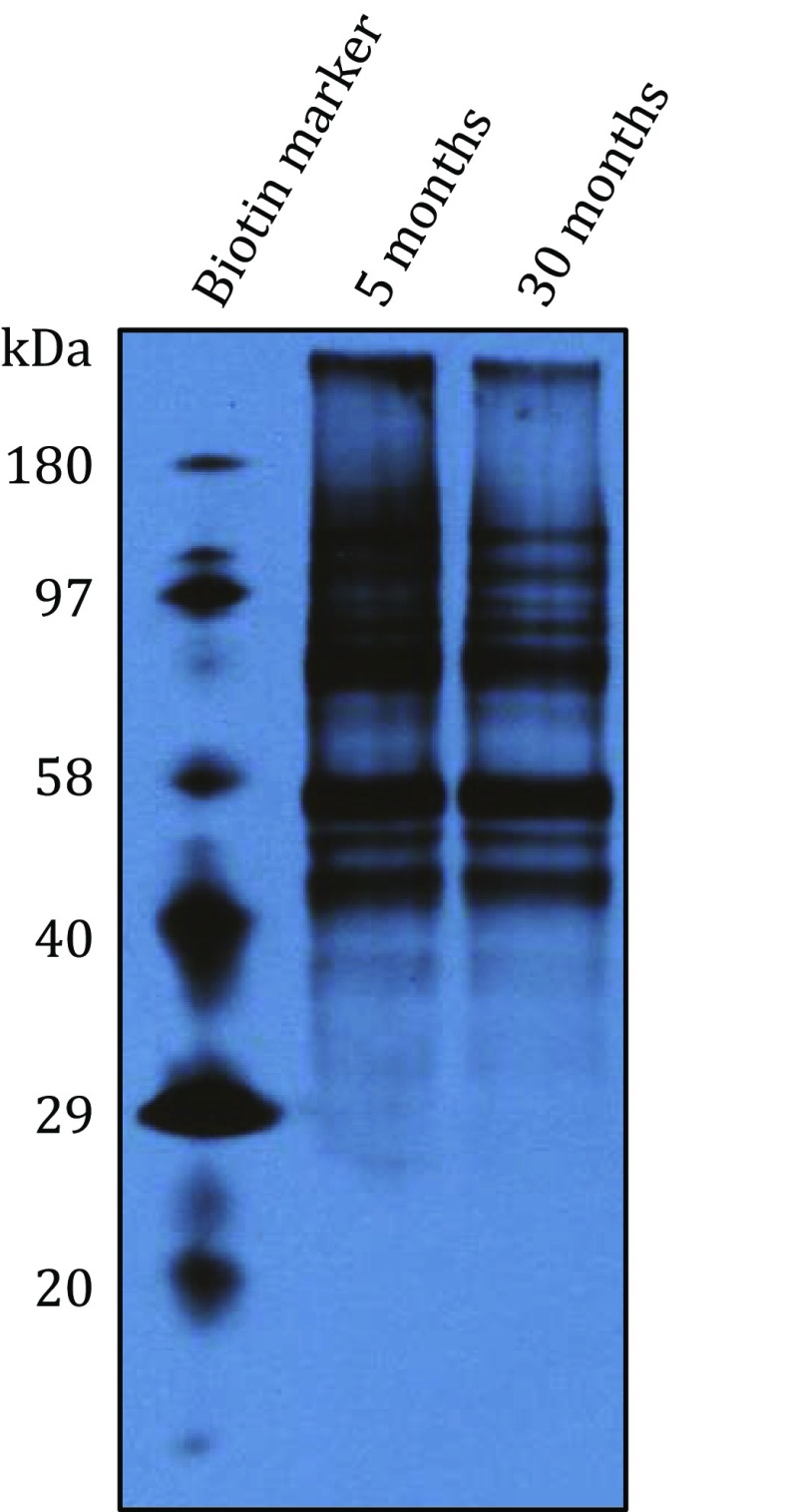
One-dimensional Western blot detection of mitochondrial protein sulfenation during brain aging. Mitochondrial proteins were treated according to the procedures presented in Fig. 1. Biotin-conjugated proteins from Bio-Rad were used as immunoblot markers

The method of arsenite reduction/biotin switch (as shown in Fig. 1) was originally applied under non-reducing electrophoretic conditions (Saurin *et al*. 2004). However, given the fact that the resolution of a reducing gel is always better than that of a non-reducing gel, we first performed one-dimensional (1D) reducing SDS-PAGE (polyacrylamide gel electrophoresis) and Western blot to test whether our procedure of trichloroacetic acid (TCA) precipitation and organic solvent washing following arsenite reduction/biotin labeling could completely remove any unincorporated biotin-maleimide that would otherwise interfere with reducing gel electrophoresis and Western blot detection. Mitochondria were oxidatively stressed *in vitro* in the presence of succinate and antimycin A (Yan *et al*. 2008) and the results are shown in Fig. 2. When the sample was treated with biotin-maleimide in the absence of sodium arsenite, no biotin signals were detected (lane 2). When the sample was treated with dimedone that specifically reacts with sulfenic acids (Saurin *et al*. 2004) before arsenite reduction and biotin labeling, no biotin signals were detected either (lane 3), indicating a complete blocking of PSOHs by dimedone. Biotin signals were only detected in the samples treated with sodium arsenite and biotin-maleimide (lane 4). Therefore, these results demonstrate that the steps of TCA precipitation and organic solvent washing used in our procedure completely removed unincorporated biotin-maleimide, and that samples treated in this way were compatible with reducing gel electrophoresis and Western blot detection of PSOHs. Nevertheless, as shown in Fig. 3, 1D Western blot analysis was not applicable in detecting age-related changes in protein sulfenation because the 1D approach yielded a very low resolution that could not produce well-defined and distinguished bands for protein identification.

### **Identification of mitochondrial proteins showing changes in cysteine sulfenation**

**Fig. 4:**
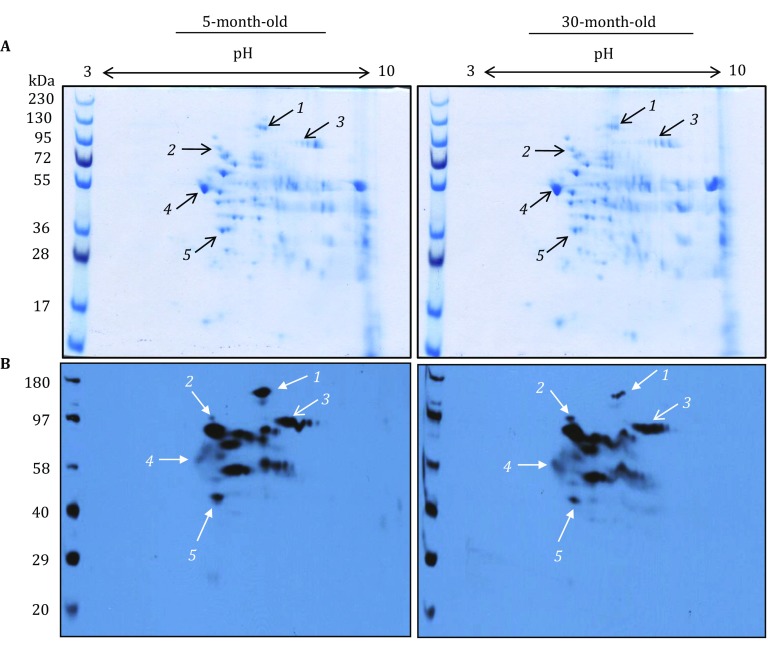
Two-dimensional gel analysis of sulfenated brain mitochondrial proteins. **A** Representative 2D maps comparing brain mitochondrial protein expression levels between 5- and 30-month-old rats. Brain mitochondria were prepared and analyzed as described in the text. Gels were stained by Coomassie blue G-250. **B** Representative 2D Western blots of age-related protein sulfenation in rat brain mitochondria. Shown are 2D gel maps of protein sulfenation in brain mitochondria isolated from 5- to 30-month-old rats (40 μg protein/gel). For these experiments, fresh mitochondria fractions were prepared followed by immediate analysis of protein sulfenic acids. Each age group contained brain mitochondria isolated from three rats

As 1D Western blot analysis didn’t work for our purpose (Fig. 3), we then took a comparative 2D Western blot approach so that comparison of sulfenation signal intensities could be made between corresponding gel spots. While there were numerous proteins on Coomassie blue-stained 2D gels (Fig. 4A), there was a less number of gel spots showing age-related changes in sulfenation when probed with HRP–streptavidin (Fig. 4B). Based on changes of the selected spot intensities between the two age groups (5 and 30 months), spots 1 and 5 showed a decrease in sulfenation; spots 2–4 showed an increase in sulfenation. These selected gel spots were then excised for MS peptide sequencing and the identified proteins are listed in Table 1, which includes pyruvate carboxylase (spot 1), aconitase (spot 3), pyruvate dehydrogenase (spot 5), mitofilin (spot 2), and tubulin α-1 (spot 4). It should be noted that no age-related changes in individual protein content could be observed between 5 and 30 months of age (Fig. 4A). Hence, any changes in sulfenation for the selected proteins were indeed due to sulfenic acid formation, rather than to changes in the levels of protein expression.

**Table 1:** Sulfenated proteins identified in each gel spot by NanoLC-MS/MS

Spots	Protein names	MW (Da)	Access (NCBI)	Number of spectral count
1	Pyruvate carboxylase	130,436.16	31543461	13
2	Mitofilin	67,477.41	77917546	16
3	Aconitase 2, mitochondrial	86,121.31	40538860	70
4	Tubulin, α-1	50,815.88	38328248	6
5	Pyruvate dehydrogenase E1	43,871.92	124430510	3

### ***In vitro*oxidative stress, protein sulfenation, and aconitase activity**

**Fig. 5:**
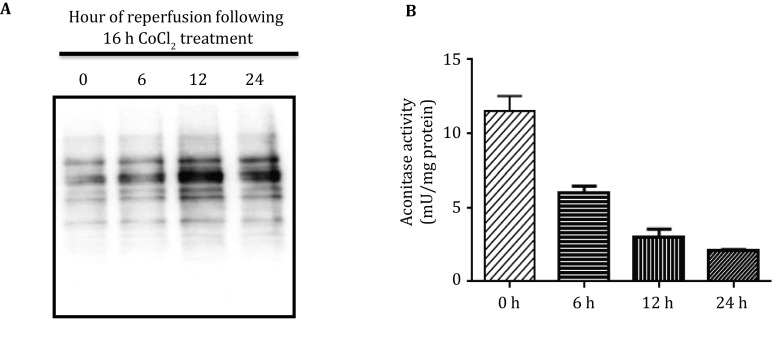
Western blot analysis of HT22 cell protein sulfenation after CoCl_2_-induced hypoxia–reperfusion. **A** Reperfusion time-dependent increase in protein sulfenation measured by DCP-Bio1 labeling and Western blot. **B** Reperfusion time-dependent decrease in aconitase activity

To investigate whether there is a relationship between overall protein sulfenation and changes in enzyme activity, we used an *in vitro* system whereby HT22 cells were treated by CoCl_2_ that is known to induce hypoxia–reperfusion oxidative stress (Sun *et al*. 2015; Wang *et al*. 2016) and then measured protein sulfenation and aconitase activity. Here PSOHs were labeled by a recently developed probe DCP-Bio1 (Klomsiri *et al*. 2010) followed by Western blot analysis. Results in Fig. 5 indicate that protein sulfenation showed an overall increase in sulfenic acid content that is reperfusion time-dependent (Fig. 5A) while aconitase activity showed a steady decrease in a reperfusion time-dependent manner (Fig. 5B). These results indicate that there was a time-dependent loss in aconitase activity in this *in vitro* oxidative stress system. While aconitase activity was inversely related to Western blot protein sulfenation profile, whether the loss of its activity is due to sulfenation to a specific cys residue on aconitase remains to be investigated. It is possible that the loss of aconitase activity could also be due to the formation of sulfinic or sulfonic acid (Han *et al*. 2005). It is also worth noting that the DCP-Bio1 method is much better and simpler than the arsenite reduction/biotin switch method as the former does not involve any washing and precipitation steps and saves time and samples. DCP-Bio1 is commercially available but is kind of expensive. For those who cannot afford to purchase this probe, the arsenite reduction/biotin switch should still be the method of choice.

## **DISCUSSION**

In the present study, we mainly used the arsenite-specific reduction/biotin switch method (Saurin *et al*. 2004) to capture PSOHs formed during brain aging. The biotin-tagged protein samples were then comparatively analyzed by 2D Western blots probed with HRP–streptavidin. Five gel spots showing age-related changes in sulfenic acid formation were excised for protein identification. Results of the present study indicate a differential nature of protein sulfenation and suggest that mitochondrial PSOHs play a role in brain oxidative stress and aging.

Given the fact that PSOHs are key intermediates during protein thiol oxidation (Poole *et al*. 2004), a decrease in a protein’s sulfenation during aging should not be surprising. When the sulfenic acids on target proteins are not stable and can undergo further oxidation, the level of protein sulfenation detected by this method would show a decrease because all those further cys oxidation products (Poole *et al*. 2004), formed via sulfenic acids, are resistant to arsenite reduction, which leads to less biotin labeling and hence a lower level of PSOH content. This is in contrast to that of protein carbonylation, an irreversible oxidative modification that occurs on multiple amino acid residues and rarely shows a decrease during aging (Yan *et al*. 1997; Yan and Sohal 1998).

Aconitase is an enzyme of the citric acid cycle. Its possession of an iron–sulfur cluster makes it not only a target susceptible to oxidative modification (Yan *et al*. 1997; Yarian *et al*. 2006), but also a sensitive marker for evaluating the magnitude of oxidative stress (Gardner 2002; Zourlidou *et al*. 2007). Aconitase not only undergoes irreversible oxidation such as protein carbonylation (Yan *et al*. 1997) and sulfenation of the cys residue that binds to the Fe–S cluster (Han *et al*. 2005), but can also experience reversible oxidative modifications such as sulfenation (this study and Charles *et al*. 2007; Saurin *et al*. 2004), S-glutathionylation, and S-nitrosylation (Han *et al*. 2005; Tsou *et al*. 2007). All these findings indicate that both irreversible and reversible modifications to aconitase can occur. Furthermore, given the findings that rat heart aconitase incurs a reversible redox inhibition upon cardiac ischemia–reperfusion (Bulteau *et al*. 2005), it’s probable that aconitase sulfenation in brain mitochondria plays a role in age-related occurrence of neurodegeneration. It should be noted that *in vitro* studies using HT22 cell treated by CoCl_2_ to mimic hypoxia–reperfusion indicate that aconitase activity showed a reperfusion time-dependent decrease (Fig. 5B) while the overall mitochondrial protein sulfenation showed a reperfusion time-dependent increase. It is likely that the decrease in aconitase activity under these experimental conditions also involves other types of protein oxidation such as carbonylation and nitrosylation (Han *et al*. 2005; Yan *et al*. 1997).

Mitofilin is an inner membrane protein that regulates mitochondrial cristae morphogenesis (John *et al*. 2005). Down-regulation of mitofilin by siRNA leads to decreased cellular proliferation and increased apoptosis due to a disorganized inner mitochondrial membrane (John *et al*. 2005). In senescence-accelerated mouse, mitofilin is implicated in cognitive disorder associated with mitochondrial dysfunction (Wang *et al*. 2008). Moreover, relevant to the pathogenesis of Parkinson’s disease, *in vitro* exposure of isolated rat brain mitochondria to reactive dopamine quinone induced a rapid loss of mitofilin protein content (van Laar *et al*. 2008), suggesting that sulfenation of mitofilin, altering the protein’s function, could be implicated in age-related neurodegenerative disorders.

Regarding sulfenation of pyruvate dehydrogenase and pyruvate carboxylase, it is expected that sulfenation of these proteins would impair mitochondrial function. Pyruvate dehydrogenase is the first component of pyruvate dehydrogenase complex that catalyzes the conversion of pyruvate to acetyl-CoA. It serves as a key link between glycolysis and the Krebs cycle. Inactivation of this protein would switch cells from aerobic to anaerobic metabolism, producing lactate instead of acetyl-CoA (Kim *et al*. 2006). Likewise, pyruvate carboxylase catalyzes the conversion of pyruvate to oxaloacetate that also feeds into the Krebs cycle (Marin-Valencia *et al*. 2010). Hence, this protein is also an important enzyme in pyruvate metabolism. On the other hand, tubulin is known to undergo cys oxidation (McDonagh and Sheehan 2007) as well as reversible glutathionylation that is most likely derived from sulfenic acid (Britto *et al*. 2005). This protein interacts with voltage-dependent anion channel (Carre *et al*. 2002; Rostovtseva *et al*. 2008) that is a part of mitochondrial permeability transition pore structure (Yan *et al*. 2002). As all these identified proteins play critical roles in mitochondrial bioenergetics, their sulfenation may collectively contribute to the overall compromise of mitochondrial respiration and oxidative phosphorylation that occur during brain aging (Vancova *et al*. 2010; Yao *et al*. 2009).

Finally, it should be pointed out that protein sulfenation during aging could also provide beneficial effects on an organism. This is because there has been increasing evidence that oxidative stress can play a positive role in the adaption to non-injurious stress (Ristow and Schmeisser 2011; Yan 2014) such as caloric restriction (Kwon *et al*. 2017) and preconditioning or postconditioning (Cohen and Downey 2011; Dost *et al*. 2008). Indeed, it has been reported that protein sulfenation can be protective against oxidative stress (Fetherolf *et al*. 2017; Mateos *et al*. 2017). Therefore, identification of protein sulfenation that is beneficial to the retardation of the aging process may provide insights into the mechanisms of beneficial redox signaling processes in fighting aging and diseases.

In summary, using comparative 2D Western blot analysis in conjunction with MS peptide sequencing, we have identified mitochondrial proteins that underwent sulfenation changes during brain aging. The alterations in the identified proteins’ sulfenic acid intensities highlight the nature of mitochondrial protein sulfenation, which can go either up or down and is likely dictated not only by the stability of the sulfenic acids in target proteins, but also by the intensity and the duration of the oxidative stress that occurs during aging. Results of this study suggest that mitochondrial protein sulfenation plays a role in mitochondrial function and/or dysfunction during brain aging. Finally, it should be alerted that sulfenation of certain cys residues in some of the identified proteins may not have any functional consequences. Hence, future studies need to identify the specific cys residues that have impacts on protein functions upon sulfenation.

## EXPERIMENTAL PROCEDURES

### **Animal and chemicals**

Male Sprague–Dawley rats were obtained from Harlan (Indianapolis, IN). Experiments were conducted in adherence with the NIH Guidelines for the Care and Use of Laboratory Animals and were approved by the University of North Texas Health Science Center Animal Care and Use Committee. All chemicals were purchased from Sigma (St. Louis, MO) unless otherwise indicated.

### **Preparation of brain mitochondria**

The isolation of whole brain mitochondria was carried out using Percoll gradient centrifugation as previously described (Yan *et al*. 2007). Brains were removed rapidly and homogenized in 15 ml of ice-cold, mitochondrial isolation buffer containing 0.32 mol/L sucrose, 1 mmol/L EDTA, and 10 mmol/L Tris–HCl, pH 7.1. The homogenate was centrifuged at 1330 *g* for 10 min and the supernatant was saved. The pellet was resuspended in 0.5 (7.5 ml) volume of the original isolation buffer and centrifuged again under the same conditions. The two supernatants were combined and centrifuged further at 21,200 *g* for 10 min. The resulting crude mitochondrial pellet was resuspended in 12% Percoll solution that was prepared in mitochondrial isolation buffer and centrifuged at 6900 *g* for 10 min. The resulting supernatant was then carefully removed by vacuum. The obtained soft pellet was resuspended in 10 ml of the mitochondrial isolation buffer and centrifuged again at 6900 *g* for 10 min. All of the mitochondrial pellets obtained after centrifugation were either used immediately or frozen at −80 °C until analysis. Protein concentrations were determined by bicinchoninic acid assay (Smith *et al*. 1985).

### ***In vitro*protein sulfenation by mitochondria-generated ROS**

Protein sulfenation in isolated brain mitochondria was induced by incubating mitochondria with succinate and antimycin A, a condition that is known to enhance mitochondrial reactive oxygen species (ROS) generation and mitochondrial oxidative stress (Turrens 1997; Turrens *et al*. 1985). Mitochondrial incubation was carried out as previously described (Schonfeld and Reiser 2006; Yan *et al*. 2008). Briefly, mitochondria (0.25 mg/mL) were incubated at 25 °C for 60 min in incubation buffer (110 mmol/L mannitol, 10 mmol/L KH_2_PO_4_, 60 mmol/L Tris, 60 mmol/L KCl, and 0.5 mmol/L EGTA, pH 7.4) in the presence of 50 μmol/L antimycin A. The mixture was then supplemented with succinate (10 mmol/L). Control samples were incubated under the same conditions in the absence of antimycin A and succinate. At the end of the incubation, mitochondria were pelleted by centrifugation at 8000 *g* for 10 min followed by labeling of PSOHs as described below.

### **Labeling of protein sulfenic acids by arsenite reduction/biotin switch assay**

The procedure used to label PSOHs, shown in Fig. 1, was performed as previously described (Saurin *et al*. 2004) with modifications. Mitochondrial pellet, immediately following isolation or *in vitro* oxidative stress challenge, was solubilized in a thiol-group blocking buffer containing 100 mmol/L sodium acetate (pH 7.0), 20 mmol/L NaCl, 1% SDS, and 100 mmol/L NEM. The protein mixture was incubated on a rotator at room temperature for 2 h followed by clarification of the mixture by centrifugation at 13,000 *g* for 10 min. Excess NEM in the supernatant was removed by gel filtration using PD-10 columns. This was followed by addition of 0.1 mmol/L biotin-maleimide and 20 mmol/L sodium arsenite (both final concentrations) to the eluate. The sample was further incubated on a rotator at room temperature for 30 min. Proteins were then precipitated by 10% TCA (final concentration) on ice for 10 min followed by centrifugation on a bench top centrifuge at 1000 *g* for 5 min. The pellet was washed three times with ethyl acetate:ethanol (1:1, *v*/*v*). Protein pellet after the third wash was used for either affinity capture or Western blot probing of the biotinylated (sulfenated) proteins.

### **Two-dimensional Western blot detection of sulfenated proteins**

2D Western blot was performed as previously described (Yan 2009) with modifications. Following biotinylation of the sulfenated cys residues, mitochondrial pellets were resuspended in 2D rehydration buffer (8 mol/L urea, 4% CHAPS, 0.2% ampholytes (pH 3–10), and 100 mmol/L DTT). First-dimensional protein separation was performed with Bio-Rad Protean IEF Cell. Samples (40 μg/IPG strip) were applied to immobilized pH gradient strips (7-cm, non-linear pH 3–10, Bio-Rad) for 1 h at room temperature. The strips were then covered with mineral oil overnight, and isoelectric focusing was performed using the preset rapid voltage ramping method. For the second dimension, the immobilized pH gradient strips were equilibrated in room temperature for 25 min in equilibration buffer (6 mol/L urea, 2% SDS, 0.05 mmol/L Tris–HCl, 20% glycerol) to which 2% DTT was added before use. An additional 25 min equilibration period was then used with the same equilibration buffer to which 2.5% iodoacetamide, instead of 2% DTT, was added. The strips were then embedded in 0.7% agarose on the top of 7.5% Laemmli polyacrylamide slab gels (no stacking gel) and run by Tricine–SDS/PAGE running buffer (Khalkhali-Ellis 1995). One of the resulting 2D gels was stained with Coomassie colloidal blue as previously described (Yan and Forster 2009), and the other gel underwent electrophoretic transfer to PVDF membrane followed by immunoblotting with HRP–streptavidin. Signals on the PVDF membrane were visualized with an enhanced chemiluminescence kit. Biotin-conjugated protein marker was used for the purpose of both molecular weight ladders and positive controls. For 1D Western blot analysis, gel electrophoresis under reducing conditions was conducted. All images were scanned by an Epson Perfection 1670 scanner.

### **Affinity capture of sulfenated proteins**

For affinity capture of sulfenated proteins that were converted to biotinylated proteins, the pellet after TCA precipitation and organic solvent washing was dissolved in 10 ml of 50 mmol/L phosphate buffer (pH 7.0) containing 150 mmol/L NaCl, 0.1% SDS, 1% Triton X-100, and 0.5% sodium deoxycholate (all final concentrations). The sample was then clarified at 13,000 *g* for 10 min and the supernatant was brought to 50 ml using the above pellet-dissolving phosphate buffer. One milliliter of high-capacity streptavidin agarose beads (Pierce, Inc.) was then added. The beads-containing sample was then rotated end-to-end for 1 h at room temperature, followed by centrifugation at 1500 *g* for 5 min. The agarose beads were then transferred to a small column, washed with 200 ml of 50 mmol/L phosphate buffer (pH 7.0) containing 1 mol/L NaCl, 0.1% SDS, 1% Triton X-100, and 0.5% sodium deoxycholate (all final concentration). The washed beads were then emptied out of the column, boiled in 0.5 ml elution buffer containing 62.5 mmol/L Tris–HCl (pH 6.8) and 1% SDS. The supernatant was then collected for analysis by Nano-liquid chromatography–mass spectrometry/mass spectrometry (LC–MS/MS).

### **Protein identification by mass spectrometry**

Protein identification was performed at ProtTech (Norristown, PA) by the LC–MS/MS peptide sequencing technology. For gel-based identification, each 2D gel spot was destained, cleaned, and in-gel digested with sequencing grade trypsin. For solution-based identification, the sample was first reduced by DTT (10 mmol/L, final concentration) and then alkylated by iodoacetamide (20 mmol/L, final concentration). Proteins were denatured by 8 mol/L urea, followed by dilution to 2 mol/L urea with 100 mmol/L ammonium bicarbonate, pH 8.5. Following trypsin digestion, the resulting peptide mixture was cleaned and analyzed by LC–MS/MS sequencing. The mass spectrometric data collected were used to search the most recent non-redundant protein database using ProtTech’s proprietary software suite; and the relative abundance of a protein in a given gel spot was determined by the corresponding spectral count number (redundant peptides and non-redundant peptides) as previously described (Liu *et al*. 2004; Roth *et al*. 2006; Vogel and Marcotte 2008). Only the proteins identified in a given gel spot that were also identified by affinity capture were reported in this study.

### **Cell culture and*in vitro*hypoxia/reperfusion treatment**

HT22 cells were cultured in DMEM (Hyclone, USA) with 10% fetal bovine serum (Hyclone, USA) and were incubated in a humidified incubator with 5% CO_2_ at 37 °C, and were seeded on 100-mm culture dishes at 100,000 cells/dish. Cell density was maintained at 80% or less confluency to attenuate excessive growth. CoCl_2_, a chemical hypoxia inducer (Ardyanto *et al*. 2006; Naves *et al*. 2013), was added into HT22 cells to develop the hypoxia model. In the hypoxia and reperfusion injury experiments, HT22 cells were incubated with 500 µmol/L CoCl_2_ for 16 h. After the removal of the culture medium, DMEM with 10% fetal bovine serum was added and maintained for 0, 6, 12, and 24 h, respectively. Control cell groups were treated with DMEM with 10% fetal bovine serum in the absence of CoCl_2_ and were maintained under the same condition. Mitochondria from these cultured cells were isolated by the same method as described above. For HT22 cellular mitochondrial sulfenic acid labeling, a DCP-Bio1 probe purchased from Kerafast (Boston, MA) was used to specifically label PSOHs (Klomsiri *et al*. 2010). Mitochondrial aconitase activities in these cells after varying treatment conditions were measured using a kit from BioAssay System (Hayward, CA) and aconitase activity was expressed at mU/mg protein as previously described (Yan *et al*. 1997).

## Abbreviations

2D-PAGE: Two-dimensional polyacrylamide gel electrophoresis
Cys: Cysteine
LC–MS: Liquid chromatography–mass spectrometry
NEM: *N*-ethylmaleimide
ROS: Reactive oxygen species
TCA: Trichloroacetic acid
